# Local plant knowledge and its variation among farmer’s families in the Napf region, Switzerland

**DOI:** 10.1186/s13002-021-00478-5

**Published:** 2021-09-03

**Authors:** Anna Poncet, Christoph Schunko, Christian R. Vogl, Caroline S. Weckerle

**Affiliations:** 1 Department of Sustainable Agricultural Systems, University of Natural Resources and Life Sciences, Vienna, Gregor Mendel Strasse 33, 1180 Vienna, Austria; 2 Department of Systematic and Evolutionary Botany, University of Zürich, Zollikerstrasse 107, 8008 Zürich, Switzerland

**Keywords:** Ethnobotany, Intracultural knowledge variation, Gender, Children, Switzerland

## Abstract

**Background:**

Local plant knowledge typically is unevenly distributed within a community. This knowledge variation is important in understanding people’s relationship with their environment. Here we ask about knowledge variation among farmers’ families in the Napf region of Switzerland.

**Methods:**

In 2008 and 2009, 60 adults and children living on 14 farms were interviewed about known and used plant species, and the data analyzed for knowledge variation. The farms were chosen by random stratified sampling, and freelisting and semi-structured interviews were conducted individually in the local idiom. The data were organized in an access database and analyzed with descriptive statistics, correlations, Mann–Whitney *U* tests and cultural domain analysis.

**Results:**

Totally, 456 folk taxa were listed, whereas frequently listed species are common meadow and forest species. Uses were indicated for 391 taxa, most of them culinary, followed by fodder, wood, medicinal and ornamental uses. Local plant knowledge correlates with age and gender. Due to professional specialization, adults above 20 years have broader plant knowledge than children and adolescents. This is true for almost all examined habitat and plant use categories except for toy uses. Women and men share a common body of plant knowledge especially about herbaceous grassland species and woody species. Specialized knowledge of men is linked to cattle fodder and the processing of wood, specialized knowledge of women concerns edible, medicinal and ornamental plants, often garden species, but also herbaceous forest species.

**Conclusion:**

In a rural region like the Napf, people retain a solid basis of plant knowledge. The variation of plant knowledge within farmers’ families of this region reflects sociocultural patterns. As these patterns are changing and as (agro)biodiversity is declining, local plant knowledge in the Napf region is suspected to undergo a mainstreaming process.

**Supplementary Information:**

The online version contains supplementary material available at 10.1186/s13002-021-00478-5.

## Introduction

Research about intracultural variation of environmental knowledge has shown that members of different social groups have different approaches to the environment and specialize in different domains of environmental knowledge [1–3]. Local plant knowledge is culturally and socially embedded and is therefore unevenly distributed within communities. Neglecting the sociocultural heterogeneity within a community may result in overlooking important parts of the knowledge and reduces therefore the accuracy of scientific findings as well as the efficiency of resulting policies [3, 4].

Many sociocultural variables, often interrelated, were found to influence the distribution of local plant knowledge. Among these figure ethnicity [1, 5], education [6–9], exposure to natural environments including homegardens [7, 10, 11], resident place [7, 11–14], income class [12], language [7] and occupation [7, 8, 11].

However, most often reported and discussed is knowledge variation due to age and gender. The two variables are often analyzed together. For example, in the community of Boumba, Niger, elderly people were found to know most about medicinal plants, and women generally know most about food plants and mid-aged men about fodder plants [1]; in the savannas of South Africa, mid-aged women and young people were found to be highly knowledgeable regarding woody plant species [13]; in the mestizo communities in Venezuela’s Caura Basin, men and older people know most about natural history of plants, while men’s and women’s knowledge about medicinal plants is equal and increases only for foreign mestizos with age [15]; in Bahia state, Brazil, women know generally more about medicinal plants than men, and for both, the knowledge increases with age [2].

The terms *sex* and *gender* are often used interchangeably in ethnobotanical literature, despite the sociological distinction between “sex” as biological characteristic and “gender” as social, cultural and psychological traits [3, 16]. In this article, we use *gender* when referring to women and men, emphasizing their role as culturally informed “knowledge-bearers.”

### Plant knowledge and gender differences

Gender is a particularly critical variable because it is linked to several other important sociocultural factors as, e.g., residence, education, occupation, income class, social status and social networks [3, 17]. Gender-based differences in plant knowledge were observed all over the world [3, 18, 19]. Women’s and men’s plant knowledge shows spatial and temporal variation, and it may differ, e.g., at the level of ecosystems, life forms (herbaceous plants, trees), folk species or plant parts [3]. While the knowledge spheres of women and men are distinct, they are also interdependent, complement one another and may also overlap [17, 19]. Some authors state that women hold greater plant knowledge and therefore greater responsibility for plant management and the maintaining of plant biodiversity than men [18], p. 7]. On the other hand, a study about medicinal plant knowledge demonstrates that on a global scale, women and men hold an equally rich knowledge, and differences become visible with smaller scale analysis only [20]. Overall, the need of carefully analyzing the situation is stressed, because the knowledge patterns are as diverse as gender roles can be.

Gendered plant knowledge is intimately linked with division of labor [3]. For family farms in Europe and the USA, a strong gender division of labor has been described [21–24; for an overview, see 25]. In short: The farmer is the owner and head of the farm; he takes the important decisions, handles the heavy machines and is responsible for the productive part, that is, the cash-generating income as crops, cattle, etc. He represents the farm, sits in agricultural organisations and makes regional politics. The farm woman is responsible for the reproductive part, that is, children, household and subsistence. As a flexible workforce, she is the assistant of her husband, especially in times of heavy workload or if the husband works off farm. In Switzerland, the farmers use most of their time for the farm work, and the farm women use half of their time for household and family and 24% for farm work. The remaining quarter is shared between other work on the farm as administration, garden or on- and off-farm occupations [26, 27]. Even in the rare cases of official female farm managers, this pattern remains almost intact [28]. As farm women are not payed for their work, their labor contribution remains invisible and is largely not recognized, neither by society and legal status nor in their own eyes [29–33]. At the same time, farm women today have multiple curriculae and growing self-esteem, there is an awakening awareness of the workforce and rights of farm women, and the numbers of female farm managers and women with a completed farmer’s formation are increasing [32–35].

### Plant knowledge and age: childrens’ plant knowledge

Children all over the world attain environmental knowledge and skills through observing, attending, imitating and helping their parents, grandparents, other adults or peers at work; all the while boundaries between work and play are fluent [36–39]. Children’s environmental knowledge is not just premature adult’s knowledge but has its own characteristics [40]. This includes special hunting techniques [40] or special snack and food gathering [1, 41].

Factors influencing children’s plant knowledge include family size, with children from larger families having broader knowledge [38]. Formal schooling was found among several indigenous societies to be of minor importance for children’s environmental knowledge or even detrimental to it, especially to practical skills [8, 9, 40–44]. In cities, biodiversity of green spaces as well as social factors such as ethnicity, economic situation, social relations and the possibility of experiencing nature independently of adults was found to influence nature knowledge of children [45].

In several countries, mainly urban areas, children were found to have alarmingly little plant knowledge: In a Swiss study, more than 6000 children and adolescents between the age of 8 and 16 years listed on average five plants out of their everyday environment [46], and the same is true for 110 students of the town Ajo, Arizona, aged 12–20 years [47]. In Germany, 25% of 1253 pupils, both urban and rural and between 11 and 14 years old, could not name a single wild-growing fruit [48]. When children from urban Brasilia were asked to draw a forest, they depicted more species if they lived near a forest, and most of the drawn species were animals, only 2.8 different plants on average [49]. Children with impoverished experience of nature develop a simplified folkbiology and an anthropocentric worldview and lack values in relation to their environment [50, 51].

### Aim of the study

This study aims to explore the state of local plant knowledge among farmers’ families of the Napf region in Switzerland. It documents plant species and associated uses as mentioned by the interviewees. The influence of gender and age as well as religion and farm management practice on plant knowledge is analyzed. The influence of religion was of interest because of a striking separation of the protestant and catholic population; and farm management practices were analyzed with respect to organic or non-organic, as more sustainable farm management systems may be linked to an enhanced interest in plants. The findings are compared with results from national and international studies on this topic.

## Methods

### Research area

**Fig. 1:**
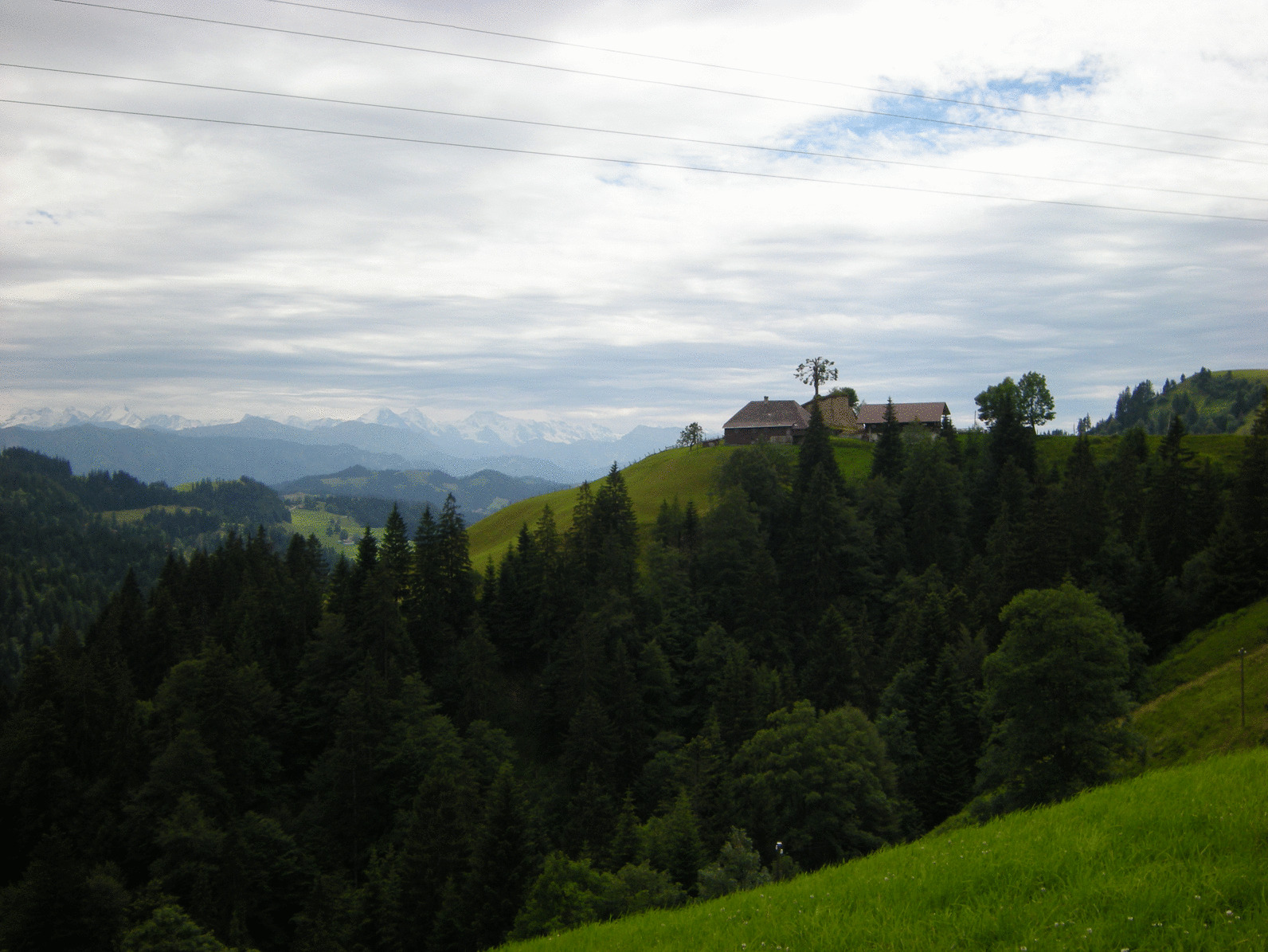
The Napf is a hilly region with solitary farms. In the background the alps (Photograph: Anna Poncet)

The Napf region is a rural region of the alpine foothills between the cities of Berne and Lucerne (Fig. 1). It is bounded by a circular valley structure, encompasses 500 km^2^ and touches 19 political communities. The border between the cantons of Berne (protestant) and Lucerne (catholic) runs across the summit of the Napf, dividing the region in two almost equal parts.

Annual precipitation amounts to 1708 mm and annual average temperature to 5.3 °C on the summit of the Napf with a height of 1400 m above sea level [52]. Height ranges down to 600 m above sea level. The underground, a molasse conglomerate, was topographically shaped by water into radially arranged valleys and ridges. The steepest and the shadowy parts are forested, whereas plainer grounds have been cleared for agriculture. Because of the difficult topography, the Napf region was populated relatively late, during the second half of the first century [53, 54]. The original form of settlement of the migrating Alemannic people is still visible: The solitary farms are surrounded by their land and a forest belt, which results in a small-scale mosaic of wood and open space; villages are restricted to the larger valleys. The forest is dominated by *Abies alba* Mill*.*, *Picea abies* (L.) H. Karst. and *Fagus sylvatica* L*.*. Meadows and pastures are rather humid and nutrient rich, often of the type *Arrhenaterion*, *Polygono-Trisetion* or *Cynosurion* [55]. Nevertheless, because of its location between the Alps and the plains, the Napf region harbors 1063 plant species [56, 57].

The traditional land use system combined arable plots (cereals and later potatoes) with pastures and meadows for cattle (cows, goats, sheep and pigs), kitchen gardens, field gardens, orchards and forest [53, 54, 58]. The Bernese part of the Napf was 50 years ago identified as a low-income, undeveloped region of the canton of Berne, and its agriculture was described as still very traditional, even “backward” [59]. However, like in most regions of Switzerland, the mechanization and the usage of fertilizer and pesticides on farms have drastically increased during the last decades. Today, agriculture in the Napf is focused on dairy farming and upbringing of young livestock, the grassland is intensively managed, and arable farming is practiced only on the lowest and plainest grounds. The intensification of agriculture lead, like everywhere in Switzerland, to a loss of biodiversity [60, 61].

A farm includes typically 10 to 20 hectares grassland plus some hectares of forest. Most farms are part-time farms with at least one person with an off-farm employment. In the nine communes lying entirely within the region, 17–73% (average 38%) of the population work in the agricultural sector, ten times more than in whole Switzerland with 3.6% [62]. The farms are usually inherited by one of the sons which leads to a patrilocal residence pattern.

### Sampling

Fieldwork was carried out by the first author during August–September 2008 and October–November 2009. A total of 60 informants living on 14 farms were interviewed. To get a balanced number of interviewees of different sociocultural variables, the farms were chosen by random stratified sampling: Out of a list of the farms, we choose randomly equal numbers of farms of the combinations Berne/organic, Berne/non-organic, Lucerne/organic and Lucerne/non-organic, respectively. We ended up working with four organic and three non-organic farms in the canton of Berne and three organic and four non-organic farms in the canton of Lucerne. On every farm, every member of the family living there was interviewed. We left aside some small kids of 4 years and below. The youngest interviewee was a boy of 8 years.

On the 14 farms, the officially registered managers are in 10 cases men, in 3 cases a couple. One couple does no longer farm. They sold the land to a neighbor, but still live on their farm, keep a garden and look after the young stock of the neighbor. In 11 of the 14 cases, the farmer has inherited the farm from his father or from another relative. On five of the 14 farms live three or even four generations together. The agricultural area of the farms (without forest) varies in size between 4.7 ha and 29.2 ha with most of them between 10 and 20 ha. All of the farmers are cattle breeders. They produce either milk or beef or they grow up young stock. Four families fatten a small number of pigs as well, three have some goats for family needs and three families keep their own bees. Additionally, on three farms cereals and potatoes are grown, on two other farms herbs for the candy company “Ricola” or the dairy “Napfmilch AG.” On all of the farms, except of one at least one home garden is managed. To every farm belongs also some forest. The avails of the sold wood can make up an important part of the income. Three farms are full-time farms; in all the other families at least one person has a paid job outside the farm, most of them part-time jobs. The jobs are done by as many women (10) as men (11).

Every member of the farmer’s family was asked for an individual interview. The interviewees comprised 33 men and boys, 8–71 years old (average 38,  ± 20.3), and 27 women and girls, 10–72 years old (average 36,  ± 19.9). Of them, 36 were living on organic and 24 on non-organic farms, and 29 were living in the canton of Berne and 31 in the canton of Lucerne.

### Data collection

Data were collected using freelists, followed by a semi-structured interview [63, 64]: The interviewees were interviewed individually and first asked to list all indigenous plants he or she could think of *(“Säg mer aui iiheimische Pflanze, wo der i Sinn chöme!”*). They were subsequently asked if the listed plants could be used for something *(“Cha me di Pflanze für öppis bruuche?”*). Prior to the interviews, the interviewees were informed about the project and asked for permission to record the interviews and to take pictures [65].

Participant observation was used by living and working on one of the farms for several weeks in 2008 and 2009, and informal conversations took place on all of the farms whenever it was possible, e.g., during meals, during working or walking together or when waiting for someone. This helped to interpret the results, especially regarding gendered labor division, knowledge acquisition of children and cultural differences between Berne and Lucerne.

The folk taxa listed by the interviewees were kept at the taxonomic level they were given. For example, “Grüen-Erle” (*Alnus viridis)* and “Erle” (*A. viridis* and *A. incana*) were interpreted as two different folk taxa. Species identification was done by means of transect walks and participant observation. Voucher specimens of forest and grassland species were taken in the presence of the informants, identified according to the Flora Helvetica [66] and deposited at the herbarium of the Natural Museum of Lucerne (NMLU, vouchers listed in [67]). Cultivated plant species of the fields and gardens were identified at the spot and photographed but not vouchered. If they do not figure in the Flora Helvetica, their nomenclature follows the publications of the Swiss edition-lmz [68–70]. We considered all species as “wild growing,” which are not sown or planted. As most of the meadows in this region are permanent grassland, meadow species were also counted as wild-growing species.

All interviews were conducted in Swiss German and were recorded and are deposited at the first author’s home. After fieldwork, each family got a summary of the results and the pictures taken at their homes.

### Data analysis

Cultural domain analysis (cultural consensus) of the freelists was performed with Anthropac to test if all interviewed persons share a basic idea about the cultural domain “indigenous plants” [71, 72].

For further analysis of the freelists, the plant reports of the freelists were assigned to different habitat and use categories. A plant report refers to one taxon mentioned by one person. The habitat categories are garden, orchard, grassland (meadow and pasture), field, forest, way- and brooksides and forest edge and other. Each taxon got only one habitat assignment; we took the habitat most often mentioned in relation with this plant. Meadow and forest were further divided into meadow grasses and meadow herbs, as well as woody, shrubby and herbaceous forest species.

Use reports of the listed plants were assigned to use categories: culinary, medicinal, ornamental, wood, toy, fodder, veterinary medicine and other (Table 1). A use report refers to one single use of a plant mentioned by one person. In the case of uses in the kitchen, we did not ask for all the different preparations. We noted if the plant is used raw (salad) or cooked (vegetable) and considered special preparations if they were mentioned spontaneously. Different levels of use specifications led to different use reports. For example, *Symphytum officinale*, besides the reported medicinal uses, got an additional use report for “the root is good for an unknown health problem.” Plant uses, which were reported to be practiced by someone else than the interviewed person, were also counted as use reports, but made discernable as that in the list of the use reports [see Additional File 1].

**Table 1:** Use categories

Culinary	The plant or parts of it is eaten raw or cooked or used for preparations like syrup, juice, herbal teas (without any medicinal indication) and alcoholic drinks
Medicinal	Uses for the medicinal treatment of humans
Ornamental	The plant is used for decoration or grown as ornamental plant
Wood	Firewood, timber, wood used for handicraft (mostly carpenter wood, but also, e.g., basket weaving) and “wood” which was mentioned without precise use
Toy	The plant is used to play with
Fodder	The plant is grazed by animals or fed to animals, including plants said to be good nectar suppliers for honeybees
Veterinary medicine	Uses for the medicinal treatment of animals
Other	E.g., herbal preparations to treat the garden plants, clover sown as fertilizer, plant characteristics used for weather forecasts, canes to herd animals, plants used in religious context etc

The variation of plant knowledge was tested using Spearman correlations and Mann–Whitney U tests. Correlations were calculated between variables indicating the level of knowledge, including freelist length, number of use reports per habitat, number of use reports per use category, and the socio-demographic variables age (in years), gender (men/women), canton (Berne/Lucerne), type of farm management (organic/non-organic). Mann–Whitney U tests were performed to compare the freelist length of children and adults (in two age groups: <  = 20 years, > 20 years) with their respective numbers of plant reports given per habitat and use category. A significance level of *p* < 0.05 was applied for identifying significant relations. All inferential statistics were calculated with SPSS 24.0.

## Results

### Known and used plant species

According to cultural domain analysis the interviewees form a quite homogeneous group, no fundamental differences between two or more groups were visible (pseudo-reliability 0.993, first eigenvalue ratio 34.121). We therefore assume that all interviewed persons share a basic idea of what belongs to the domain “indigenous plants.”

The 60 freelists contained 7 to 108 items (arithmetic mean 44.6,  ± 26.5). In total, 456 folk taxa of different folk-taxonomical ranks were listed, including 14 fungi. Of these, 214 taxa are wild growing, 43 are wild growing but also cultivated, 191 are cultivated on fields or in homegardens, and 8 could not be identified. The folk taxa were assigned to 425 species, subspecies and cultivars and additional 32 genera (for more details, see [67]).

Families with more than 10 listed folk taxa were Asteraceae (47), Rosaceae (41), Poaceae (40), Lamiaceae (35), Brassicaceae (24), Fabaceae (24), Apiaceae (15), Ranunculaceae (14) and Amaryllidaceae (13).

The most frequently listed species is *Taraxacum officinale*, followed by *Rumex obtusifolius* and *Rubus fruticosus.* The 20 most often listed taxa by all interviewees are very common species of pastures and forests, mostly wild growing. An exception is the cultivated fruit trees, apple and pear (*Malus domestica*, *Pyrus communis*), and linden tree (*Tilia cordata*, *T. platyphyllos*). Table 2 shows the differences between the most often mentioned species among women, men and children.

**Table 2:** The 20 most often listed folk taxa by all participants (*n* = 60), women (*n* = 16), men (*n* = 24) and children and adolescents (*n* = 20, 9 boys and 11 girls), ordered according times mentioned

Scientific name	Times mentioned	Women (*n* = 16)			Men (*n* = 24)			Children and adolescents (*n* = 20, 9 boys and 11 girls)	All participants (n=60)	Scientific name	Times mentioned
		German name	Scientific name	Times mentioned	German name	Scientific name	Times mentioned	German name	German name		
*Taraxacum officinale*	52	Schwarzer Holunder	*Sambucus nigra*	14	Löwenzahn	*Taraxacum officinale*	22	Löwenzahn	Löwenzahn	*Taraxacum officinale*	16
*Rumex obtusifolius*	43	Löwenzahn	*Taraxacum officinale*	14	Blacke	*Rumex obtusifolius*	20	Sonnenblume	Blacke	*Helianthus annuus*	12
*Rubus fruticosus* agg	36	Blacke	*Rumex obtusifolius*	14	Rotklee	*Trifolium pratense*	18	Apfelbaum	Brombeere	*Malus domestica*	11
*Abies alba*	34	Brombeere	*Rubus fruticosus* agg	14	Weissklee	*Trifolium repens*	18	Ahorn	Weisstanne	*Acer pseudoplatanus*	10
*Rubus idaeus*	33	Himbeere	*Rubus idaeus*	13	Knaulgras	*Dactylis glomerata*	15	Blacke	Himbeere	*Rumex obtusifolius*	9
*Malus domestica*	33	Hahnenfuss	*Ranunculus acris, R. repens*	13	Englisch Raygras	*Lolium perenne*	14	Weisstanne	Apfelbaum	*Abies alba*	9
*Sambucus nigra*	33	Weisstanne	*Abies alba*	12	Spitzwegerich	*Plantago lanceolate*	14	Birnbaum	Schwarzer Holunder	*Pyrus communis*	9
*Urtica dioica*	33	Margrite	*Leucanthemum vulgare*	12	Apfelbaum	*Malus domestica*	14	Linde	Brennessel	*Tilia cordata/T*	9
*Picea abies*	33	Rotklee	*Trifolium pratense*	12	Buche	*Fagus sylvatica*	14	Brennessel	Rottanne	*Platyphyllos*	9
*Acer pseudoplatanus*	32	Weissklee	*Trifolium repens*	12	Ahorn	*Acer pseudoplatanus*	13	Gras	Ahorn	*Urtica dioica*	9
*Trifolium pratense*	32	Rottanne	*Picea abies*	12	Brombeere	*Rubus fruticosus* agg	13	Brombeere	Rotklee	*Poaceae*	9
*Trifolium repens*	32	Spitzwegerich	*Plantago lanceolate*	11	Rottanne	*Picea abies*	13	Eiche	Weissklee	*Rubus fruticosus* agg	9
*Plantago lanceolate*	31	Heubeere	*Vaccinium myrtillus*	11	Hasel	*Corylus avellana*	13	Rottanne	Spitzwegerich	*Picea abies*	8
*Fagus sylvatica*	29	Schlüsselblume	*Primula elatior, P. veris*	11	Brennessel	*Urtica dioica*	13	Himbeere	Buche	*Rubus idaeus*	8
*Ranunculus acris / R. repens*	29	Brennessel	*Urtica dioica*	11	Birnbaum	*Pyrus communis*	13	Zwetschgenbaum	Hahnenfuss	*Prunus domestica*	8
*Pyrus communis*	29	Vogelbeere	*Sorbus aucuparia*	11	Weisstanne	*Abies alba*	13	Schwarzer Holunder	Birnbaum	*Sambucus nigra*	7
*Tilia cordata/T. platyphyllos*	28	Buche	*Fagus sylvatica*	10	Timothegras	*Phleum pratense*	12	Farn	Linde	*Polypodiaceae*	7
*Leucanthemum vulgare*	28	Thymian	*Thymus serpyllum*	10	Esche	*Fraxinus excelsior*	12	Gänseblümchen	Margrite	*Bellis perennis*	7
*Corylus avellana*	26	Hasel	*Corylus avellana*	10	Margrite	*Leucanthemum vulgare*	12	Hahnenfuss	Hasel	*Ranunculus acris / R. repens*	7
*Primula elatior, P. veris*	26	Salbei	*Salvia officinalis*	9	Schwarzer Holunder	*Sambucus nigra*	12	Klee	Schlüsselblume	*Trifolium* spp.	7

**Fig. 2:**
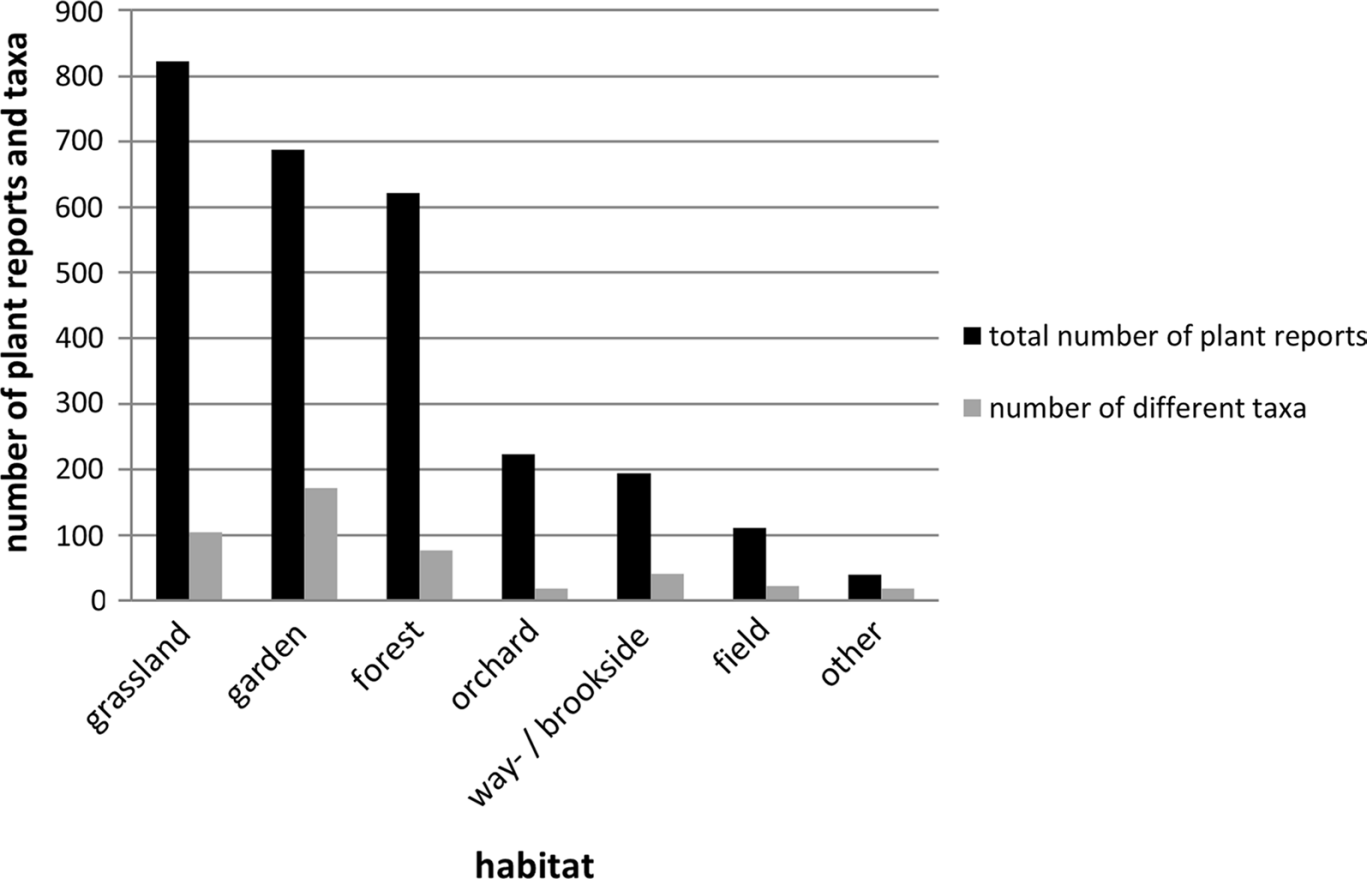
Number of plant reports (black) and taxa (grey) per habitat (*n* = 60)

The interviewees favored grassland taxa (823 plant reports), followed by garden (688) and forest (621) (Fig. 2). Garden contains greatest taxa diversity (172), followed by grassland (104), forest (77), way- and brookside (41), field (22), orchard (19) and other (13).

In total, 3335 use reports were recorded. By far, the most use reports were given for culinary uses (1310 use reports), followed by fodder (510), wood (429), medicinal uses (390), ornamental uses (330), toy (110), veterinary medicine (58) and other uses (198) (Fig. 2). Most often named culinary uses were pears (*Pyrus communis*, 26) and apples (*Malus domestica*, 24) as fruits to eat raw and the fruits of *Sambucus nigra* for jam and jelly (25); most often named fodder plants were *Taraxacum officinale* (35), *Trifolium pratense* (29) and *Trifolium repens* (28); most often named woody uses are *Picea abies* (22), *Fagus sylvatica* (22) and *Abies alba* (21) as firewood as well as *Picea abies* (19) and *Abies alba* (18) as most important timber wood; most often named medicinal uses were herbal tea of the flowers of *Primula elatior*/ *veris* (13) in case of colds, especially cough, and the syrup of the fruits of *Sambucus nigra* (11) poured in hot herbal tea against cough; most often named ornamental uses are mosses (Bryophyta, 11) and twigs of *Ilex aquifolium* (11) for decoration and the flowers of *Leucanthemum vulgare* (11) for bouquets; most often named toy uses were the flowers of *Bellis perennis* (8) worked into garlands and wreathlets; and the most often named use report for veterinary medicine is to fix twigs of *Berberis julianae* (3) on the ceiling of the stable against eczemas.

**Fig. 3:**
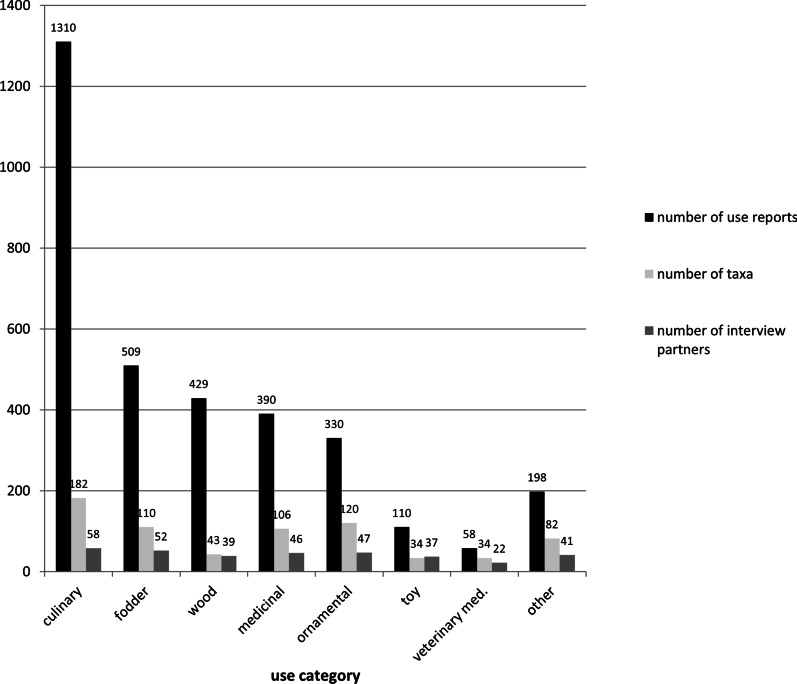
Number of use reports (black) and taxa (light grey) per use category and number of interviewees (grey) who mentioned uses in the respective use category (*n* = 60)

Use reports were given for 391 taxa [see Additional file 1]. For 55 taxa, also listed in the additional file, no use report was given. The culinary use category contained most taxa (182), followed by ornamental (120), fodder (110), medicinal (106), wood (43), toy and veterinary medicine (both 34) (Fig. 3). The interviewees were most aware of culinary uses, which were mentioned by all of them except for two.

### Knowledge differences

**Fig. 4:**
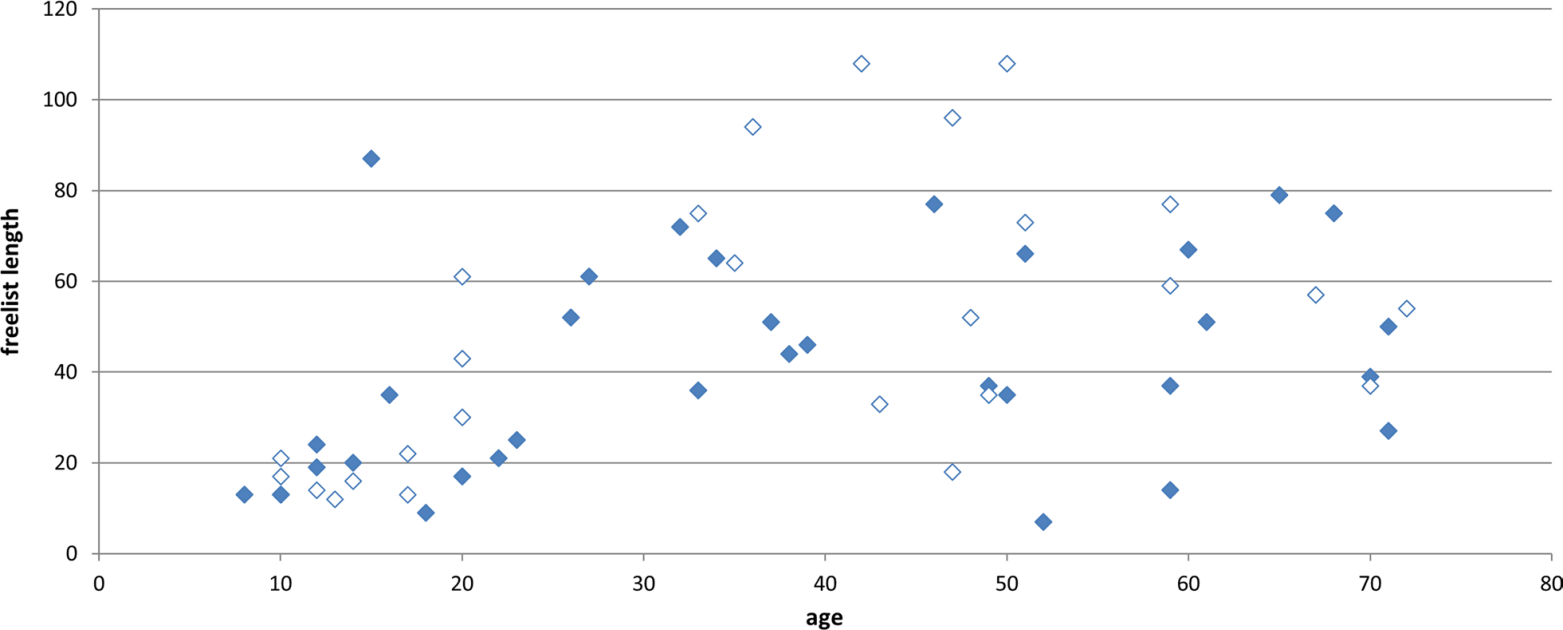
Freelist length and age of the interviewees (*n* = 60). Filled spots represent lists of male, white spots of female interviewees

The length of the freelists correlates with age (*r* = 0.490, *p* < 0.001) (Fig. 4). As children and adolescents seem to form a separate group with shorter freelists, we repeated the Spearman correlation with only the adults above 20 years (*n* = 40). Among adults, freelist length does no longer increase with age (*r* = − 0.024, *p* = 0.882). Children and adolescents up to 20 years (*n* = 20) listed less plants (mean 24.95, ± 18.72) than interviewees above 20 years (*n* = 40, mean 54.35, ± 24.29) (Mann–Whitney *U* test: *p* < 0.001).

Besides having much shorter freelists than adults, children and adolescents were additionally unevenly distributed between organic and non-organic farms. The correlations between socio-demographic variables and freelist length, habitat and use categories were therefore tested only for adults above 20 years (*n* = 40, Table 3). We detected almost no differences in freelist length, nor in habitat or use category of the listed plants between the interviewed adults of different age, from organic and non-organic farms, or the protestant canton of Berne and the catholic canton of Lucerne. There was only a weak correlation of veterinary plant uses and Lucernese interviewees (*r* = 0.370, *p* = 0.019), and elder interviewees listed slightly more species growing on way- and brooksides (*r* = 0.365, *p* = 0.021).

**Table 3:** Spearman correlations of gender, age (in years), farm management and canton of the interviewed adults (*n* = 40) with freelist length, habitats and use reports of different categories

Freelist length		Freelist length							
Gender	*r*_spearman_	− 0.310							
(1 = female, 2 = male)	*p*	0.052							
Age	*r*_spearman_	− 0.024							
(in years)	*p*	0.882							
Management	*r*_spearman_	− 0.171							
(1 = organic, 2 = non o.)	*p*	0.290							
Canton	*r*_spearman_	− 0.189							
(1 = Berne, 2 = Lucerne)	*p*	0.243							

Habitat		Garden	Orchard	Field	Grassland	Forest	Way-/brookside	Other sites	
Gender (1 = female, 2 = male)	*r*_spearman_	− .439^**^	0.047	0.007	0.024	− 0.237	− 0.270	− .326^*^	
	*p*	**0.005**	0.774	0.966	0.881	0.141	0.092	**0.040**	
Age (in years)	*r*_spearman_	− 0.070	− 0.213	− 0.077	0.021	0.034	.365^*^	0.129	
	*p*	0.670	0.186	0.639	0.899	0.834	**0.021**	0.427	
Management (1 = organic, 2 = non o.)	*r*_spearman_	− 0.181	0.055	0.048	− 0.100	0.000	− 0.140	− 0.089	
	*p*	0.265	0.737	0.767	0.540	1.000	0.389	0.586	
Canton (1 = Berne, 2 = Lucerne)	*r*_spearman_	− 0.085	0.206	− 0.085	− 0.243	− 0.104	− 0.101	− 0.113	
	*p*	0.603	0.203	0.601	0.130	0.522	0.537	0.486	

Special habitat		Grassland: grasses	Grassland: herbs	Forest: tree	Forest: shrub	Forest: herbaceous			
Gender (1 = female, 2 = male)	*r*_spearman_	.495^**^	− 0.288	− 0.084	− 0.255	− .408^**^			
	*p*	**0.001**	0.071	0.605	0.112	**0.009**			
Age (in years)	*r*_spearman_	− 0.061	0.056	− 0.017	0.042	0.119			
	*p*	0.706	0.730	0.916	0.798	0.466			
Management (1 = organic, 2 = non o.)	*r*_spearman_	0.042	− 0.176	0.092	− 0.075	− 0.166			
	*p*	0.799	0.277	0.574	0.647	0.306			
Canton (1 = Berne, 2 = Lucerne)	*r*_spearman_	− 0.046	− 0.272	− 0.087	− 0.090	− 0.114			
	*p*	0.778	0.090	0.593	0.581	0.485			

Use category		Edible plants	Fodder	Medicinal plants	Ornamental plants	Wood	Toy	Vetmed	Other uses
Gender (1 = female, 2 = male)	*r*_spearman_	− .533^**^	.365^*^	− .583^**^	− .425^**^	0.190	− .423^**^	0.066	− 0.022
	*p*	**0.000**	**0.020**	**0.000**	**0.006**	0.241	**0.007**	0.686	0.891
Age (in years)	*r*_spearman_	− 0.184	0.016	0.279	− 0.146	− 0.204	− 0.155	0.187	− 0.291
	*p*	0.254	0.922	0.081	0.367	0.207	0.339	0.247	0.068
Management (1 = organic, 2 = non o.)	*r*_spearman_	− 0.143	0.039	− 0.167	0.074	0.109	− 0.245	− 0.111	− 0.280
	*p*	0.378	0.811	0.302	0.649	0.504	0.128	0.496	0.080
Canton (1 = Berne, 2 = Lucerne)	*r*_spearman_	0.065	− 0.172	0.059	− 0.017	− 0.153	0.012	.370^*^	− 0.241
	*p*	0.690	0.290	0.719	0.915	0.346	0.943	**0.019**	0.135

Bold values indicate *p* < 0.05)

Gender turned out as relevant factor for the distribution of plant knowledge among adults. Women listed more garden plants (*r* = 0.439, *p* = 0.005), herbaceous forest species (*r* = 0.408, *p* = 0.009) and plants of different small habitats like ponds or flowerpots (*r* = 0.326, *p* = 0.040). They gave more use reports for edible plants (*r* = 0.533, *p* = 0.000), medicinal plants (*r* = 0.583, *p* = 0.000), ornamental plants (*r* = 0.425, *p* = 0.006) and plants used for toy uses (*r* = 0.423, *p* = 0.007). Men gave slightly more use reports for fodder plants (*r* = 0.365, *p* = 0.020). With respect to the amount of listed grassland plants, there are no differences visible at first glance. When dividing the grassland plants in herbs and grasses, however, men listed significantly more meadow grasses than women (*r* = 0.495, *p* = 0.001). The man who listed most grasses, a trained farmer, listed 17 species. The farm woman who listed most grasses listed 7 species and is a trained ecologist. Children and adolescents listed no other grass taxa than summary names like “Gras” and “Schmäle,” describing vaguely all Poaceae and many Cyperaceae and Juncaceae.

Adults gave higher numbers of plant reports than children and adolescents for the habitats “field,” “meadow/pasture,” “forest,” “way-/brookside,” “meadow grasses,” “meadow herbaceous plants,” “forst tree,” “forest shrub,” “forest herbaceous,” and for the use categories “edible plants,” “fodder plants,” “medicinal plants,” “veterinary medicine” and “other uses” (Mann–Whitney *U* test,Table 4). Children and adolescents gave more reports only in the use category “toy.” No differences were visible in the habitat categories “garden,” “orchard” and “other sites” as well as in the use categories “wood” and “ornamental.”

**Table 4:** Differences between children/adolescents (< = 20 years) and adults (> 20 years) in numbers of plant reports per freelist, habitat category and use category (Mann–Whitney U test)

Variable	Age groups	*n*	Arithmetic mean	SD	*p* value
Freelist length	Children/Adol.(< = 20 years)	20	24.95	19.204	0.000
	Adults(> 20 years)	40	54.35	24.603	
Garden	Children/Adol.(< = 20 years)	20	8.00	8.974	0.194
	Adults(> 20 years)	40	13.20	15.604	
Orchard	Children/Adol.(< = 20 years)	20	3.15	2.700	0.354
	Adults(> 20 years)	40	4.00	3.080	
Field	Children/Adol.(< = 20 years)	20	0.80	2.016	0.011
	Adults(> 20 years)	40	2.38	3.364	
Meadow/pasture	Children/Adol.(< = 20 years)	20	5.35	3.438	0.000
	Adults(> 20 years)	40	17.90	8.366	
Forest	Children/Adol.(< = 20 years)	20	5.95	4.828	0.002
	Adults(> 20 years)	40	12.55	8.515	
Way- or brookside	Children/Adol.(< = 20 years)	20	1.55	1.701	0.000
	Adults(> 20 years)	40	4.10	2.925	
Other sites	Children/Adol.(< = 20 years)	20	0.50	0.761	0.681
	Adults(> 20 years)	40	0.75	1.149	
Meadow (grasses)	Children/Adol.(< = 20 years)	20	0.60	0.598	0.000
	Adults(> 20 years)	40	4.43	4.119	
Meadow (herbaceous)	Children/Adol.(< = 20 years)	20	4.75	3.354	0.000
	Adults(> 20 years)	40	13.48	6.081	
Forest (trees)	Children/Adol.(< = 20 years)	20	3.15	3.392	0.014
	Adults(> 20 years)	40	6.38	4.996	
Forest (shrubs)	Children/Adol.(< = 20 years)	20	0.60	0.821	0.000
	Adults(> 20 years)	40	2.60	2.193	
Forest (herbaceaous)	Children/Adol.(< = 20 years)	20	2.20	2.913	0.043
	Adults(> 20 years)	40	3.58	3.129	
Edible plants	Children/Adol.(< = 20 years)	20	14.25	13.603	0.021
	Adults(> 20 years)	40	25.50	19.396	
Fodder	Children/Adol.(< = 20 years)	20	3.65	3.870	0.000
	Adults(> 20 years)	40	11.03	7.495	
medicinal plants	Children/Adol.(< = 20 years)	20	2.45	3.706	0.000
	Adults(> 20 years)	40	8.83	8.149	
Ornamental plants	Children/Adol.(< = 20 years)	20	3.15	3.703	0.112
	Adults(> 20 years)	40	6.63	8.136	
Wood	Children/Adol.(< = 20 years)	20	4.75	8.052	0.199
	Adults(> 20 years)	40	8.43	8.930	
Toy	Children/Adol.(< = 20 years)	20	2.25	2.124	0.033
	Adults(> 20 years)	40	1.58	2.640	
Veterinary medicine	Children/Adol.(< = 20 years)	20	0.00	0.000	0.000
	Adults(> 20 years)	40	1.38	1.944	
Other uses	Children/Adol.(< = 20 years)	20	1.55	2.704	0.003
	Adults(> 20 years)	40	4.15	4.458	

## Discussion

### Known and used plant species

Compared to regional ethnobotanical studies, our number of 391 used folk taxa is relatively high. Examples of comparable studies from Europe [39, 73–75] and other continents [14, 76–82] report between 126 and 448 used species.

**Fig. 5:**
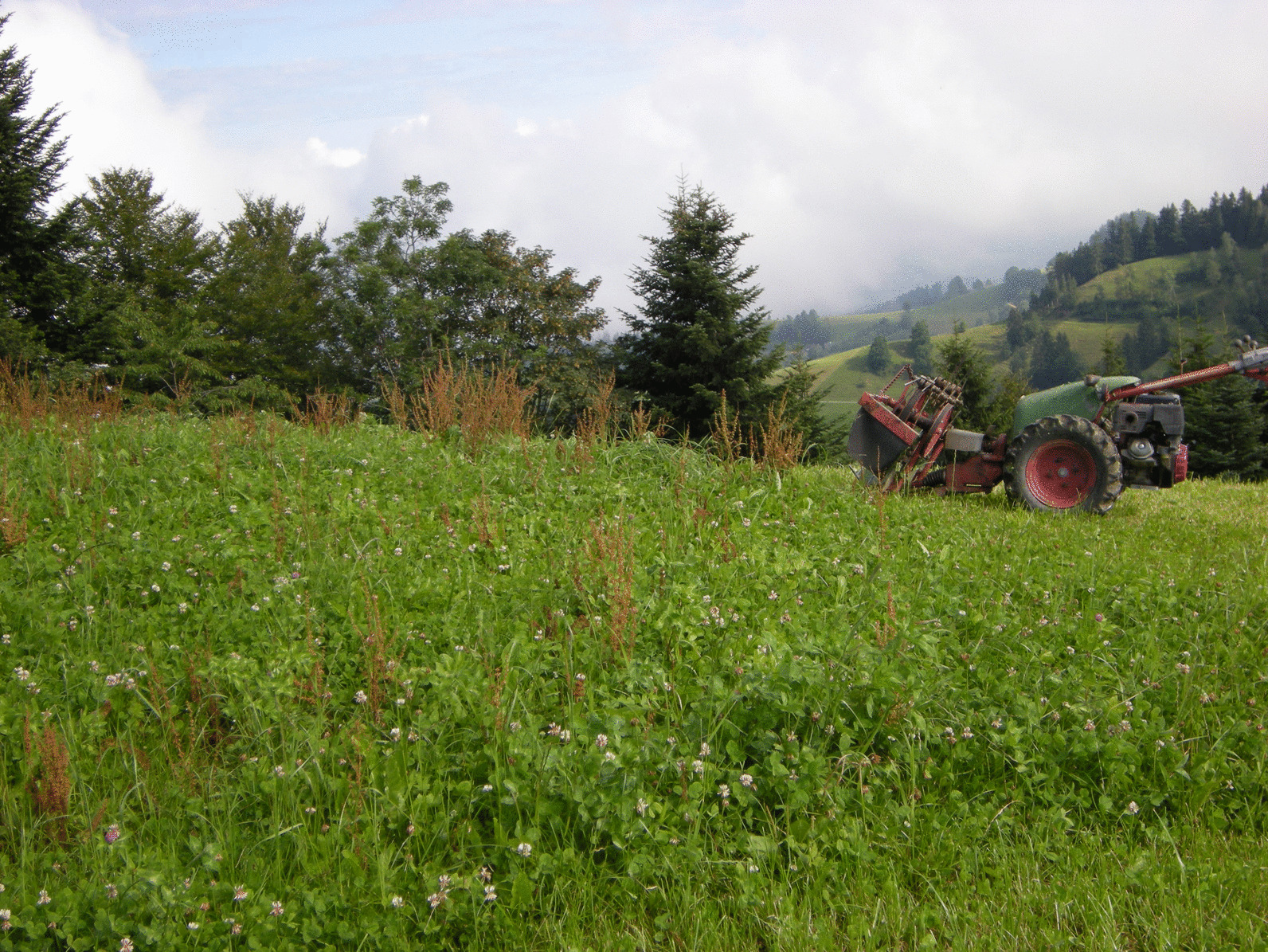
A meadow with as many “Blacke” (*Rumex obtusifolius*, red seed heads) can only be found in the canton of Lucerne (Photograph: Anna Poncet)

Dandelion (*Taraxacum officinale*) appears as most popular plant of the region, listed on top by men and women as well as children. It is very abundant on the rich pastures and meadows and reported as a fodder herb and medicinal plant, leaves and flowers are used in the kitchen, children use it for different games, and it is also a weed in gardens. Wherever this cosmopolitan weed grows [83], it is well known and cited as medicinal and edible plant in ethnobotanical surveys, e.g., in the Indian Himalaya [84], Georgia/Caucasus [85], Spain [86], Cameroon [87], South Africa [88], Mexico [78], USA [89 for native North Americans]. In a Swiss study, where school children were invited to highlight plants and animals on their way to school, the dandelion was the most often chosen species [90, p. 667]. The second placed *Rumex obtusifolius* is mostly known as a very persistent, bothersome weed on pastures in the Napf region. Its handling is time-consuming and moreover different in the cantons of Berne and Lucerne, respectively. Grassland without the large, easily visible *Rumex obtusifolius* is the pride of Bernese people, which sneeze at the lazy Lucernese farmers who let too much “Blacke” grow on their pastures. On the other hand, Lucernese people make fun of the nit-picking Bernese people, who want to have everything neat and clean (Fig. 5). In this light, *Rumex* management appears as a means to reinforce identity and to delimit the cultural borders between the catholic and protestant population.

Not only *Taraxacum officinale* and *Rumex obtusifolius* but all of the 13 taxa mentioned by more than half of the interviewees are very common species and also all related to practical experience (use, control or both). Weeding especially leads to intimate knowledge of a species. Many cultivated plants were once weeds, and many weeds are used and even managed: As readily available species with fast reproduction and an enhanced probability to contain bioactive compounds, they are discussed in the literature as shifting in a continuum between spontaneous and cultivated plants [91–93]. In our study, 53 (14%) of the taxa with reported uses were also mentioned as weeds. For only 21 (28%) of the 74 weeds, no use report was given.

### Variations of plant knowledge

Age and gender appeared as relevant factors for knowledge variation.

### Age

In the Napf region, basic plant knowledge is acquired during childhood and adolescence, which is demonstrated by the 25 taxa listed on average by children and adolescents. Consensus analysis shows that although the childrens’ freelists were shorter, they were not considerably different from the lists of the adults.

#### Children’s freelists

Many of the 20 most often listed species are the same as for the adults. Taxa only named by children show some typical features of taxa, which are known to be learned first: folk generic taxa as *Gras*, *Klee* and *Farn* and big, salient species as the trees [38, 47, 94, p. 63]. Sunflower, *Helianthus annuus*, is a very salient species which was listed by 60% of the children but only by 25% of the adults. Its sheer size makes it impressive and hard to miss in a garden.

Overall, the lists of the children and adolescents were very divers and contained only few species in common. Besides the very open freelist question, which can overstrain especially young children, we see two main possible reasons for this finding. First, children and adolescents from eight to twenty years are actually a very heterogeneous group. Secondly, unlike the adults, children have no generally defined sphere of activity at home. Their job is to go to school and perform there as good as possible. They may help at home, but are not forced to work. As far as we observed, personal interests as well as the parents and grandparents motivation and attitude regarding environmental knowledge seem to be important factors influencing the children’s plant knowledge. This would be in accordance with other studies [43].

#### Children’s specialities in plant knowledge

As mentioned in other studies [1, 41], snack foods like chewing sorrel (*Rumex acetosa*) or sucking clover flowers (*Trifolium pratense*) were also reported by our interviewees, mainly children. But as most prominent use category of the Napf children emerged “toy,” that is, all sorts of ludic activities with plants. The spectrum is large (from oracles, teasing each other and “jewellery” to waterpipes and blowing away dandelion seeds) and in its essence known from other ethnobotanical studies in Europe, either explicitly in special chapters [39, 73, 95] or implicitly [96]. Not only named the children significantly more “toy” use reports than adults, but also women significantly more than men. This reflects the traditional gender roles on farms where children, especially small ones, are surveyed by women.

#### Children’s knowledge acquisition

While the children gave short freelists compared to the adults, the amount of listed plants is high compared to the numbers reported from educational studies in urban regions of Switzerland and other countries. Intuitively, it seems quite plausible that farmer’s children know more about plants than urban children, because we imagine them surrounded of natural environments like fields, gardens, pastures and forests and going every day long ways to school through this landscape, experiencing nature at many occasions independently from adults. But “simply being outside does not make one absorb knowledge about local plants” [47], p.5], because the acquisition of differentiated folkbiological knowledge has a strong social component. Unlike most urban children, the Napf children have the possibility to participate in daily work activities of their parents and often also other adults living on the farm like grandparents or apprentices, and “work” means in this case to an important part interaction with the natural environment.

In the Napf region, formal education begins for children at the age of four to six years. During the following years, focusing on advancement in school, they will miss at home the important “key transition in the development of expertise occurring between the ages of five and nine years […], representing the time at which children in agrarian economies are quickly integrated into the family’s work activities” [36, p. 379, 42]. While in certain indigenous societies, the knowledge of children is comparable to adults from 11 years onwards [36, p. 379], our data suggest that local plant knowledge in the Napf region does not considerably increase during school years. Accelerated increase in knowledge around 20 years indicates that young adults begin to take over responsibility on the farm and have to catch up the required knowledge.

#### Knowledge differences of adults of different age

The only statistical difference in plant knowledge between adults of different age is the tendency of elders to list more species of “way- and brooksides.” The unfertilized way- and brooksides are habitats, where many species can be found, which formerly were more frequent in the grassland, as *Hypericum* spp., *Origanum vulgare*, *Geum rivale* or *Eriophorum* spp. While elder interviewees saw them every day, younger interviewees are less acquainted with them. The bias may thus be a consequence of biodiversity loss in the agricultural landscape. This suggests that a frequent and close contact between humans and plants is required to obtain and preserve plant knowledge (see also [97, p. 124]). In Switzerland, ecologically specialized species (e.g., from dry, wet or alpine habitats) are constantly disappearing and replaced by generalist species like dandelion [98, p. 12]. We assume that this “homogenization” of the Swiss Flora also leads to homogenized plant knowledge.

### Gender

Men and women in the Napf region share a common body of plant knowledge especially about herbaceous grassland species and woody species. However, the influence of gendered division of labor became clearly visible in specialized plant knowledge.

#### Men and grasses

In the Napf region, cattle breeding is the main income source and lies in the responsibility of men. It is reflected in their specific knowledge about fodder species, especially grass species. They observe closely the quality of the grassland, because it is of crucial importance for the business. When speaking about grass species, they distinguish good grass species and bad grass species with respect to their quality as fodder and give indications about where and how they grow on their land. *Dactylis glomerata*, *Lolium perenne* and *Phleum pratense*, three valuable fodder grasses, figure among the most often cited species by men. In contrast to other meadow species like dandelion (*Taraxacum officinale*), clover (*Trifolium* spp.) or daisies (*Leucanthemum vulgare*, *Bellis perennis*), the knowledge of grass species requires an extra effort given the difficulties of their identification. This explains why women and children, who are not directly involved, named less specific names but more summary names like “grass.”

The 24 interviewed men are a relatively well-defined group, all of them (with one exception) grew up in the region, they are almost all farmers and 17 of them went through the same agricultural formation. Farmers with no formal education tended to list lower numbers of grasses and less specific names, whereas all the 12 men who listed more than six grass species were formally educated farmers. Two young farmers mentioned that they would not recognize all the listed grasses in the meadow, but that they remember their names from their classes. During the interviews, many men tried hard to recall the formal knowledge, and it was presented as the “correct” knowledge. Its knowledge-uniforming influence is obvious and certainly intended by agricultural schools and advisory services.

#### Species of the forest: a mixed picture

Forest and timber are also perceived as male domain. Woodcutting and processing are almost exclusively men’s work, done during the winter months. Sold wood is an important income source. Men gave detailed use reports for different kinds of wood. For example, dividing walls in the piggery are made of ash tree, oak was chosen for a new staircase, and shingles or cheese shelves are made of spruce. However, compared to culinary use reports, woody use reports were less detailed. This bias may be due to the fact that the interviewer was a woman.

No difference was found among the number of tree species mentioned by men, women and children. This can be explained by the salience of tree species which are easily perceived, and early learned [38, 47]. Women mentioned also woody tree uses, but mostly as fire or construction wood in a more unspecific way which explains that no statistical difference was found between men and women for the use category “wood.” Additionally, women mentioned tree use as “food,” “ornamental” and “medicinal.” This is true not only for the fruit trees in the orchards, but also for the forest trees like spruce (*Picea abies*) and fir (*Abies alba)*, which play an important role in the Napf region [97, p. 114]. They yield the most valuable wood in this region, but many other uses were mentioned, mainly by women [for examples, see Additional file 1]. Women also listed more names and uses for herbaceous forest species, as berries and small flowers for example (we counted berries like black- or blueberries as herbaceous species, because they are no shrubs in the eyes of non-botanists). They rendered a broader view of the forest, compared with men, who spoke of the forest as basically made of trees. Timber production and processing as a male domain and the use of NTFPs (non-timber forest products) as a mostly female domain is an often described pattern of gendered plant knowledge [17, p. 3, 18].

#### Women’s plant knowledge

**Fig. 6:**
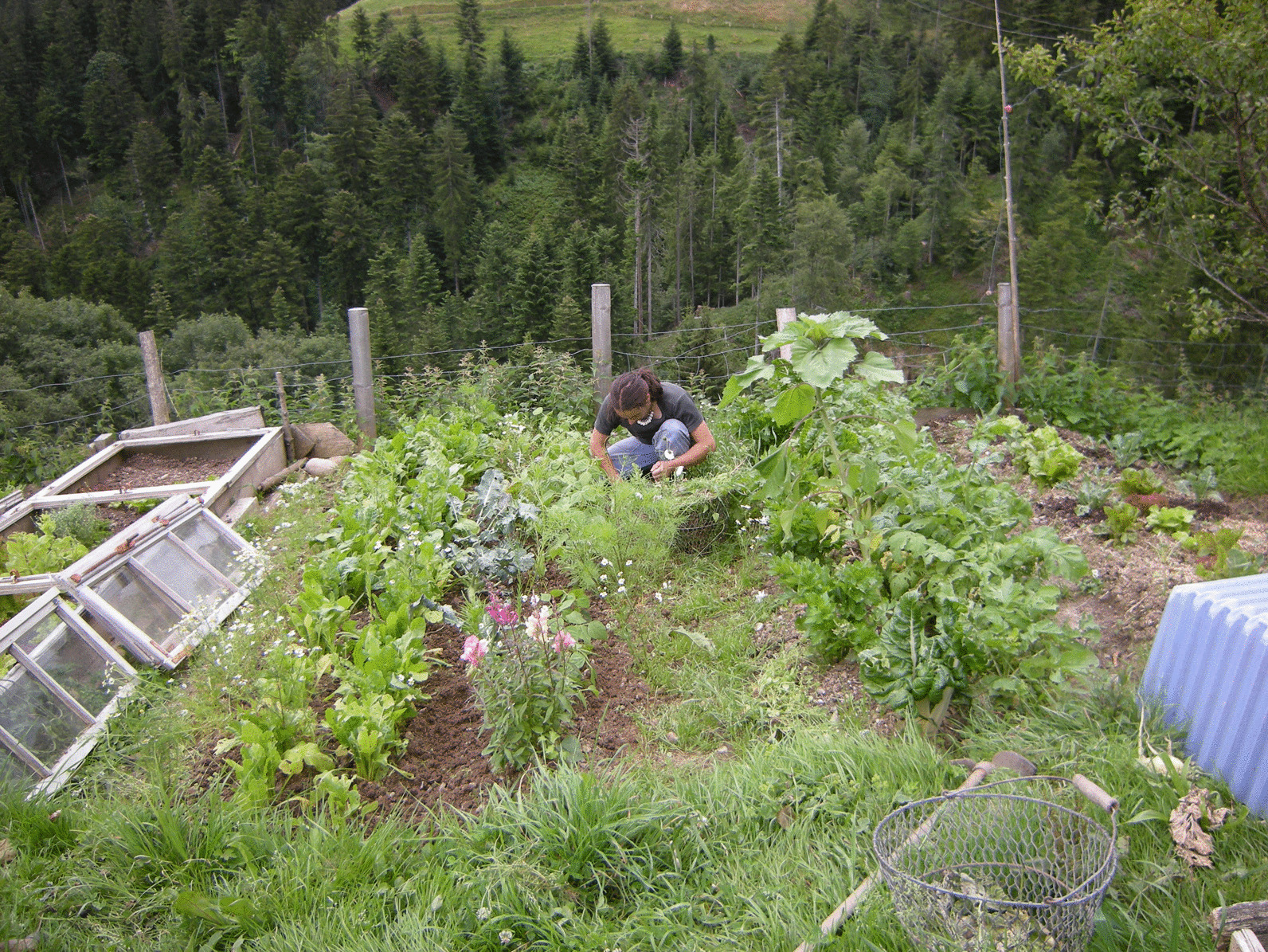
Homegardens are largely a women’s domain (Photograph: Anna Poncet)

Special plant knowledge of women embraces edible, medicinal and ornamental plants. While the women use many wild-growing species, a female specialty is the home garden (Fig. 6).

Usually, the farmer’s wife is responsible for the garden. If her mother-in-law is still living on the farm, the two women have each her own garden. Vegetables, berries, herbs, medicinal and ornamental plant species are grown, almost exclusively for the needs of the family. The high importance of home gardens in the Napf region as representational space for farm women was underlined in several studies and has been shown also for other regions in Switzerland [35, p. 109, 59,99,100]. Only one of the farms in our study had no garden any more. Due to her off-farm work, the woman had not enough time to present her garden as nice as she would have liked and was ashamed of the people of the near village and the hikers passing by.

Interestingly, there are only three garden species found among the 20 most often listed plants among women: Sage (*Salvia officinalis*), blackberry and raspberry (*Rubus fruticosus*, *R*. *idaeus*), whereas the two latter names also include the wild forms and figure among the most often cited plants of men too. This indicates that women in total listed many garden plants—but all of them listed slightly different species. This suggests a considerable variability between the respective gardens.

In Eastern Tyrol, the differences of the floristic composition of homegardens were explained with “individual patterns of plant use” [101, p. 361]. This also applies for our region. Women’s plant knowledge has heterogeneous origins. While a majority of the married women (10 out of 17) grew up on a farm, most of them learned and performed a profession outside agriculture. Only one has the official diploma “Bäuerin”; all the others became farm women by marriage. According to the patrilocal residence pattern, the women come moreover from many different places; 10 of the 17 married women are from outside the Napf region. Therefore, their gardening skills are rarely acquired through professional training but influenced by mothers, mothers-in-law, friends, neighbors, books, courses, etc. While men, through professional education, agricultural policy and the needs of the market, tend to focus on the same special type of management or crop, women seem to be less constrained and freer in their scope. This is also underlined by the abovementioned study [101], which shows that home gardens on organic and non-organic farms do not differ in their management and floristic composition.

### Other sociocultural variables

In the statistical analysis, neither the type of farm management nor the religious affiliation turned out to be important for plant knowledge. But one striking difference in plant use between Bernese and Lucernese concerns the religious custom of preparing a “*Palme*” (palm) for Palm Sunday, the Sunday before Easter. The catholic people in the canton of Lucerne prepare a bunch of different twigs from usually seven species: *Ilex aquifolium*, *Buxus sempervirens*, *Juniperus communis*, *Juniperus sabina*, *Taxus bacchata*, *Pinus sylvestris* and *Corylus avellana*. This “*Palme*” is consecrated at the mass and then taken home, where it is supposed to protect the farm and its inhabitants from evil, especially thunderstorms. In the protestant canton of Berne, most people ignore “*Palmen*” completely. More in-depth interviews about personal relations to religion and nature would be necessary to work out the background of such differences. Overall, it seems that the outlines of catholic and protestant areas are getting blurred. As several of the families demonstrated, it is, for example, no longer a problem to cross the border when marrying.

### Methodological remarks

Freelisting and subsequent semi-structured interviews proved to be a fast and simple approach to elicit knowledge about plant species and plant uses. Even if no interviewee can recall on the spot all the plants he or she knows, the outcome gives a good idea about generally known plants and individual emphasis. Supplementary information gathered during transect walks, informal interviews and participant observation embedded the listed taxa in a context and allowed, e.g., the identifications of taxa, which were listed with a short description instead of a name (e.g., “very thistly thistles” meaning *Cirsium vulgare*).

Still, the exam-like setting of a freelisting interview was in some cases observed to have a stressing effect on the interviewee, and the very open freelist question “all indigenous plants” was quite overwhelming. With such a broad domain, “people tend to omit some items and cluster responses as they unpack mental subcategories” [102]. An example for such a mental subcategory are the trees, which were sometimes forgotten or even deliberately left out, because they are not in the first instance associated with the notion „plant “. Actually, for our interviewees, a “plant” meant first of all a herbaceous plant.

Freelisting was especially difficult and probably too abstract for younger children. Asking for narrower domains like “trees” or “meadow plants” would not only produce more exhaustive lists [102] but would also facilitate to gain data from children, especially when complemented with other child friendly methods as child-guided interviews in the vicinity of their farm [41].

## Conclusions

Plants appear as immanent part of peoples’ life in the Napf region, with different life conditions shaping their perception and management. All the while we observe a gendered plant knowledge according to the traditional roles of women and men on farms, these roles are slowly changing. The re-defining of gender roles on farms goes hand in hand with a decline in homegardens, mentioned for Switzerland in general (Swiss Statistics 2013 in [33, p. 9]), and also noticed on the visited farms in the Napf region: During our studies between 2008 and 2012, the big homegardens have disappeared on 5 of the 14 farms. The loss of this agrobiodiversity will likely be followed by the loss of the respective plant knowledge of farm women and will reduce the knowledge differences between women and men. A similar reduction of local plant knowledge of younger adults due to biodiversity loss in the grassland is expected. Overall, a mainstreaming process of plant knowledge is expected: People will still know plants and related uses, but as (agro)biodiversity erodes, the focus will be on common knowledge about common species.

The influence of formal and informal learning on children’s plant knowledge needs further attention. We suspect that individual experience made at home is more important than formal schooling. It is also of interest how the tension between contradicting aspects of informal and formal knowledge of farmers is solved. Furthermore, the observed cultural differences between the Bernese and Lucernese population would merit an in-depth study. It seems that different customs with catholic background favor a spiritual attitude towards plants, which was less perceptible on the protestant side of the Napf.

## Supplementary Information

**Additional file 1.** List oft the species and reported uses.

## Data Availability

The data supporting the results are presented in the tables of the article and in the additional file. More details can be requested of the corresponding author.
